# Aqueous-phase catalytic hydroxylation of phenol with H_2_O_2_ by using a copper incorporated apatite nanocatalyst

**DOI:** 10.1039/c9ra02021g

**Published:** 2019-05-07

**Authors:** Abdallah Amedlous, Othmane Amadine, Younes Essamlali, Karim Daanoun, Mina Aadil, Mohamed Zahouily

**Affiliations:** VARENA Center, MAScIR Foundation, Rabat Design, Rue Mohamed El Jazouli Madinat El Irfane 10100-Rabat Morocco m.zahouily@mascir.com; Laboratoire de Matériaux, Catalyse et Valorisation des Ressources Naturelles, URAC 24, FST, Université Hassan II-Casablanca Morocco

## Abstract

Copper incorporated apatite (Cu-apatite) nanomaterial was prepared by a co-precipitation method. The obtained material was characterized by thermogravimetric analysis (TGA), X-ray diffraction (XRD), X-ray photoelectron spectroscopy (XPS), Fourier transform infrared (FT-IR) and Raman spectroscopy, scanning electron microscopy (SEM, STEM) and nitrogen adsorption–desorption. The as-prepared Cu-apatite was used to catalyze phenol hydroxylation with hydrogen peroxide as an oxidant. The influencing parameters including reaction time, temperature, H_2_O_2_/phenol ratio and catalyst mass have been investigated. Under the optimized conditions, the Cu-apatite catalyst gave a phenol conversion of 64% with 95% selectivity to dihydroxybenzenes. More importantly, the results of catalyst recycling indicated that the same catalytic performance has been obtained after four cycles with a slight loss of activity and selectivity.

## Abstract 2

Copper incorporated apatite (Cu-apatite) nanomaterial was prepared by a co-precipitation method.

## Graphical abstract

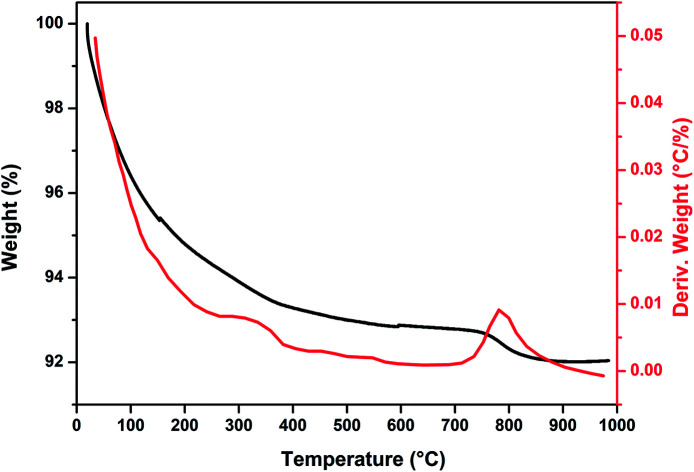

## 1. Introduction

It is well known that phenol and its derivatives are considered as hazardous pollutants due to their high toxicity even at low concentrations, which causes toxicity to human beings and animals. Indeed, phenol and phenolic compound removal from wastewater represents an important area of research, especially with the new increasing stringent requirement of environmental laws.^1^ In recent years, phenol hydroxylation using hydrogen peroxide as an oxidant has received much attention due to its efficiency to convert phenol to high value phenolic compounds (catechol (CAT) and hydroquinone (HQ)), which are widely used as chemicals in the pharmaceutical industry, polymerization inhibitors, photography chemicals, antioxidants and cosmetic products.^2,3^ Hydroxyl radicals (OH˙) produced from hydrogen peroxide are considered as a strong oxidant with a high redox potential, and can react with many organic compounds. The activation of H_2_O_2_ to produce OH˙ can be achieved by different ways, including the use of Fe^2+^ and/or Co^2+^ salt (used in the Brichima process),^4^ strong mineral acid (such as H_3_PO_4_ or HClO_4_ employed in the Rhone–Poulenc process),^5^ and a Fenton's reagent (used in the Hamilton process).^6^ However, most of them have multiple disadvantages including the use of toxic solvents, high temperature reaction, and product contamination with metal ions, which limit their industrial applications. Several attempts have been devoted to overcome these limitations and to improve phenol hydroxylation process using heterogeneous solid catalysts. However, phenol hydroxylation over heterogeneous catalysts has received an increasing attention due to its easy separation from the products, which makes its recycling and its reusability simple. Though various types of heterogeneous catalysts have been studied for this reaction, copper based catalyst have been fascinating due to its precise OH˙ radical activating including mesoporous silica Cu/MCM-41,^7^ organic frameworks Cu-MOFs,^8^ alumino-phosphate molecular CuAPO-11 (ref. 9) and mixed oxide CuO@CeO_2_.^10^

**Scheme 1:**
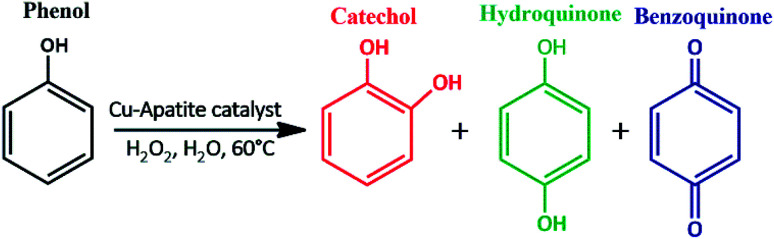
Phenol hydroxylation catalyzed by copper incorporated apatite (Cu-apatite).

Recently, apatite-based materials have attracted increasing interest due to its interesting properties which are needed in a wide range of potential application in pharmaceuticals, biomedical, and water treatment.^11,12^ These materials showed various advantages such as, high adsorption capacity, acid–base properties, high dispersion in aqueous phase, non-toxicity, chemical and thermal stability, and abundantly available in nature.^13,14^ In addition, apatite can act as catalysts by themselves or as supports for other active phases due to their excellent ion-exchange capacity where the divalent calcium ions are substituted by other metal ions. Moreover, apatite has been widely used as catalyst or support catalyst for various chemical transformations namely, C–C homo-coupling reaction,^15^ reduction of nitric oxide with ammonia,^16^ N-arylation of imidazoles,^17^ biodiesel production,^18^ synthesis of naphthopyran derivatives^19^ and cycloaddition reactions.^20^ To the best of our knowledge, the use of copper incorporated apatite as a heterogeneous catalyst for the phenol hydroxylation in water has not been reported in the literature. Accordingly, in continuation of our efforts to explore a green and performing nanocatalyst, we were planning to prepare copper incorporated apatite and investigate its catalytic performance on phenol hydroxylation with H_2_O_2_ as an oxidant under optimal conditions (Scheme 1). Furthermore, the structural, textural, surface and morphological properties of the prepared nanocatalyst, the influence of reaction temperature, phenol : H_2_O_2_ molar ratio, solvent and catalyst mass were investigated. The reusability and leaching of the catalyst were also evaluated.

## 2. Experimental

### 2.1 Materials and apparatus

The chemical reagents used in this study including calcium nitrate Ca(NO_3_)_2_·4H_2_O, copper nitrate Cu(NO_3_)_2_·3H_2_O, ammonium phosphate (NH_4_)_2_HPO_4_, ammonium hydroxide NH_4_OH 28–30%, phenol, and 30 wt% aqueous H_2_O_2_ were purchased from Aldrich Chemical Company. All chemicals were analytical grade and were used directly without any further purification. X-ray diffraction (XRD) patterns of the catalysts were obtained at room temperature on a Bruker AXS D-8 diffractometer using Cu-Kα radiation in Bragg–Brentano geometry (*θ*–2*θ*). SEM and STEM micrographs were obtained on a Tecnai G2 microscope at 120 kV. The gas adsorption/desorption data was collected using a Micromeritics 3Flex Surface characterization analyzer, using nitrogen. Prior to nitrogen sorption, all samples were degassed at 200 °C overnight. The specific surface areas were determined from the nitrogen adsorption/desorption isotherms (at −196 °C), using the BET (Brunauer–Emmett–Teller) method. Pore size distributions were calculated from the N_2_ adsorption isotherms with the “classic theory model” of Barret, Joyney and Halenda (BJH). Fourier transforms infrared (FT-IR) spectra of samples in KBr pellets were measured on a Bruker Vector 22 spectrometer. The copper content of Cu-apatite materials was determined by a flame atomic absorption spectrometer A flame atomic absorption spectrometer (AA-7000), equipped with an optical double-beam photometric system is automatically set for flame measurements, and the high-throughput, single-beam photometric system is automatically set for furnace measurements. EDS spectra from the single points and chosen microareas and maps showing elemental composition and surface distribution were obtained using the SEM-EDS method. NMR solid-state experiments were recorded on a Bruker Avance 600 spectrometer. The Raman spectra of the catalyst were recorded on a DXR™ 2 Raman Microscope (ThermoFisher Scientific) with a backscattering mode at room temperature in the range 400–1200 cm^−1^. The samples were excited using 532 nm laser with a resolution of 4 cm^−1^. To determine the surface compositions of catalyst synthesis, X-ray photoelectron spectroscopy (XPS) analysis was performed with Shimadzu Co: AXIS ULTRA. The X-ray source was Mg Kα operated at 15 kV, the anodic current was 10 mA, and the operating pressure in the vacuum chamber was lower than 5 10^−7^ Pa.

### 2.2 Preparation of copper-incorporate apatite (Cu-apatite)

Cu-apatite was synthesized using co-precipitation method according to the method reported in the literature^21^ with few adjustments. In a typical procedure, Ca(NO_3_)_2_·4H_2_O (9.446 g, 0.04 mol) and Cu(NO_3_)_2_·3H_2_O (2.416 g, 0.01 mol) were dissolved in 100 mL of deionized water and stirred to form a clear solution. Subsequently, 100 ml of (NH_4_)_2_HPO_4_ (0.3 M) was added into the prepared solution under continuous stirring ((Ca + Cu)/P molar ratio was kept constant at 1.67), followed by the addition of NH_4_OH to adjust until a pH of 10–11. The resulting mixture was heated at 90 °C for 12 h under continuous stirring. The blue precipitate was collected by centrifugation, washed several times with deionized water and ethanol, and then dried overnight at 80 °C for 12 h in hot vacuum desiccator. Finally, the as-obtained product was calcined at 700 °C for 3 h.

### 2.3 Phenol hydroxylation over Cu-apatite catalyst

Phenol hydroxylation was carried out in a flask of 50 mL equipped with magnetic stirrer and a reflux condenser. In a typical procedure, 0.47 g of phenol (5 mmol) and 0.05 g of Cu-apatite catalyst were added into 10 ml of deionized water, followed by added drop-wise 1 ml of H_2_O_2_ (10 mmol) to the mixture (The molar ratio of phenol : H_2_O_2_ was 1 : 2). The reaction temperature was subsequently heated up to 60 °C under vigorous stirring for 2 hours. At the end of the reaction, the catalyst suspensions were removed and the product was analysed and quantified using high-performance liquid chromatography (HPLC Shimadzu Kyoto, Japan) equipped with a reversed-phase C18 column (150 mm × 4.6 mm × 5 μm) using the methanol/water ((40/60) volume ratio) as mobile phase at a flow rate of 0.5 mL min^−1^ with UV detection at 190 nm. The degree of phenol conversion and the products selectivity were calculated using the following equation:

Conversion of phenol:1
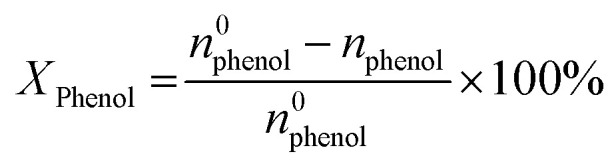


Selectivity of catechol:2
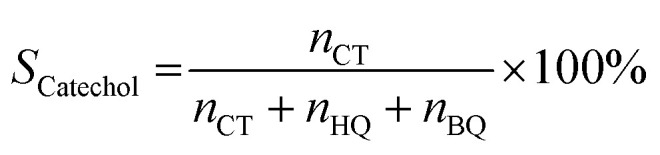


Selectivity of hydroquinone:3
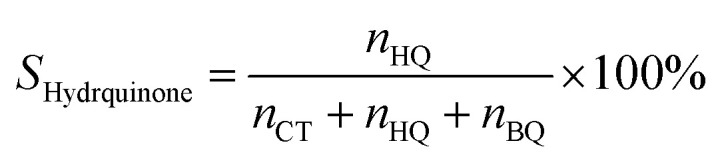


Selectivity of benzoquinone:4
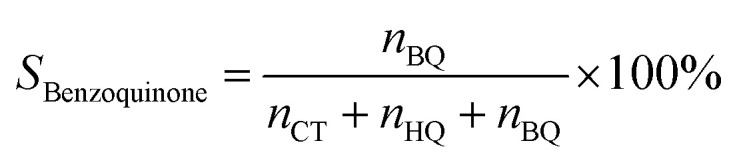
where *n*^0^_phenol_ and *n*_phenol_ are the initial and the final amounts (moles) of phenol, respectively. Whereas *n*_CT_, *n*_HQ_ and *n*_BQ_ are the produced amounts (moles) of catechol, hydroquinone and benzoquinone, respectively.

### 2.4 Cu-apatite catalyst reusability

The reusability of Cu-apatite in the phenol hydroxylation was investigated. After one catalytic test, the Cu-apatite was separated by centrifugation and washed with water and dichloromethane several times, and calcined at 700 °C for 3 h. The regenerated Cu-apatite catalyst was reused for the next run.

## 3. Results and discussion

### 3.1 Catalysts characterization

**Fig. 1:**
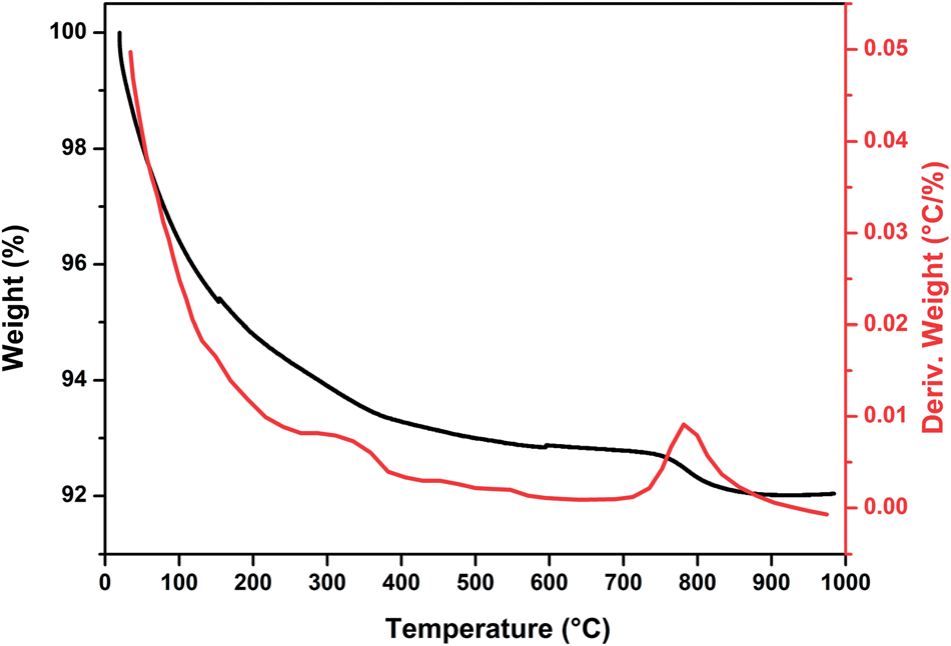
TGA curve of dried Cu-apatite under air atmosphere.

Fig. 1 show thermogravimetric analysis curve of copper incorporated apatite obtained by co-precipitation method. According to the TG curve of the Cu-apatite dried sample, two weight losses steps were observed. The first weight loss occurs from 100 to 150 °C, and can be attributed to the loss of water molecules adsorbed on the surface of Cu-apatite.^22,23^ The second weight loss from 180 to 650 °C can be related to the precursor decomposition and the elimination of structural water from Cu-apatite. Similar results have been reported by Suresh Kumar *et al.*^24^ In addition, a weight loss at 800 °C was observed, which can be attributed to the decomposition of apatite structure to form Ca_3_(PO_4_)_2_ and CaO.^25^

**Fig. 2:**
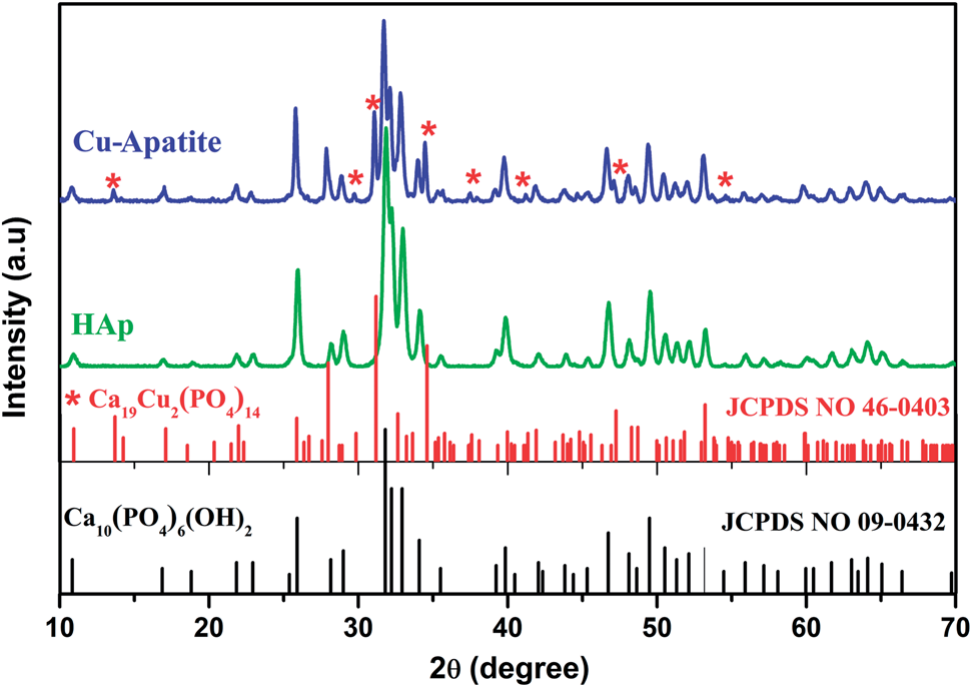
XRD patterns of HAp and Cu-apatite samples.

**Scheme 2:**
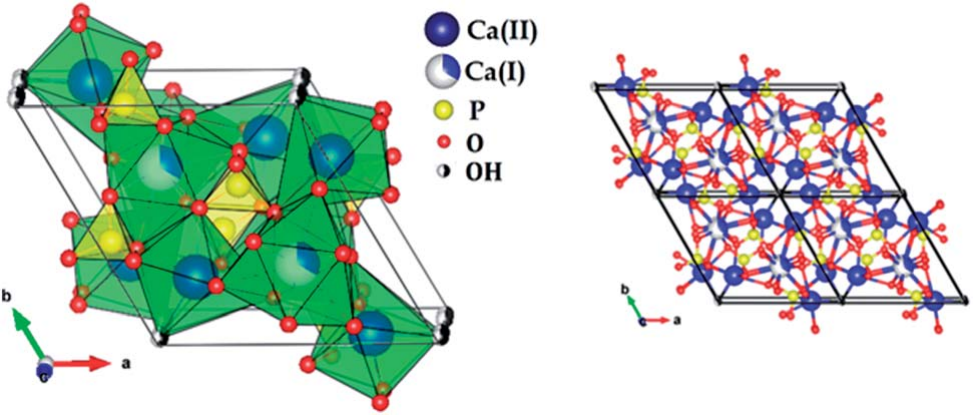
Structural representation of hydroxyapatite.

Fig. 2 shows the X-ray diffraction patterns of the hydroxyapatite, and copper incorporated apatite prepared by co-precipitation method. Accordingly, the XRD patterns of HAp (Fig. 2) confirmed the presence of peaks corresponding to hexagonal structure Ca_10_(PO_4_)_6_(OH)_2_ (space group *P*6_3_/*m* (176); JCPDS card no. 09-0432), where the Ca^2+^ at site I are connected to 9 oxygen atoms, while Ca^2+^ at site II are connected to only 5 oxygen atoms and 1 hydroxyl group (Scheme 2). Moreover, there are some remaining Ca(i) and Ca(ii) exposed on the surface of the HAp, which can be easily substituted. On the other hand, the diffraction patterns of Cu-apatite samples showed the presence of hydroxyapatite and a secondary phase in which the diffraction peaks correspond to Ca_19_Cu_2_(PO_4_)_14_ (JCPDS 46-0403).

**Fig. 3:**
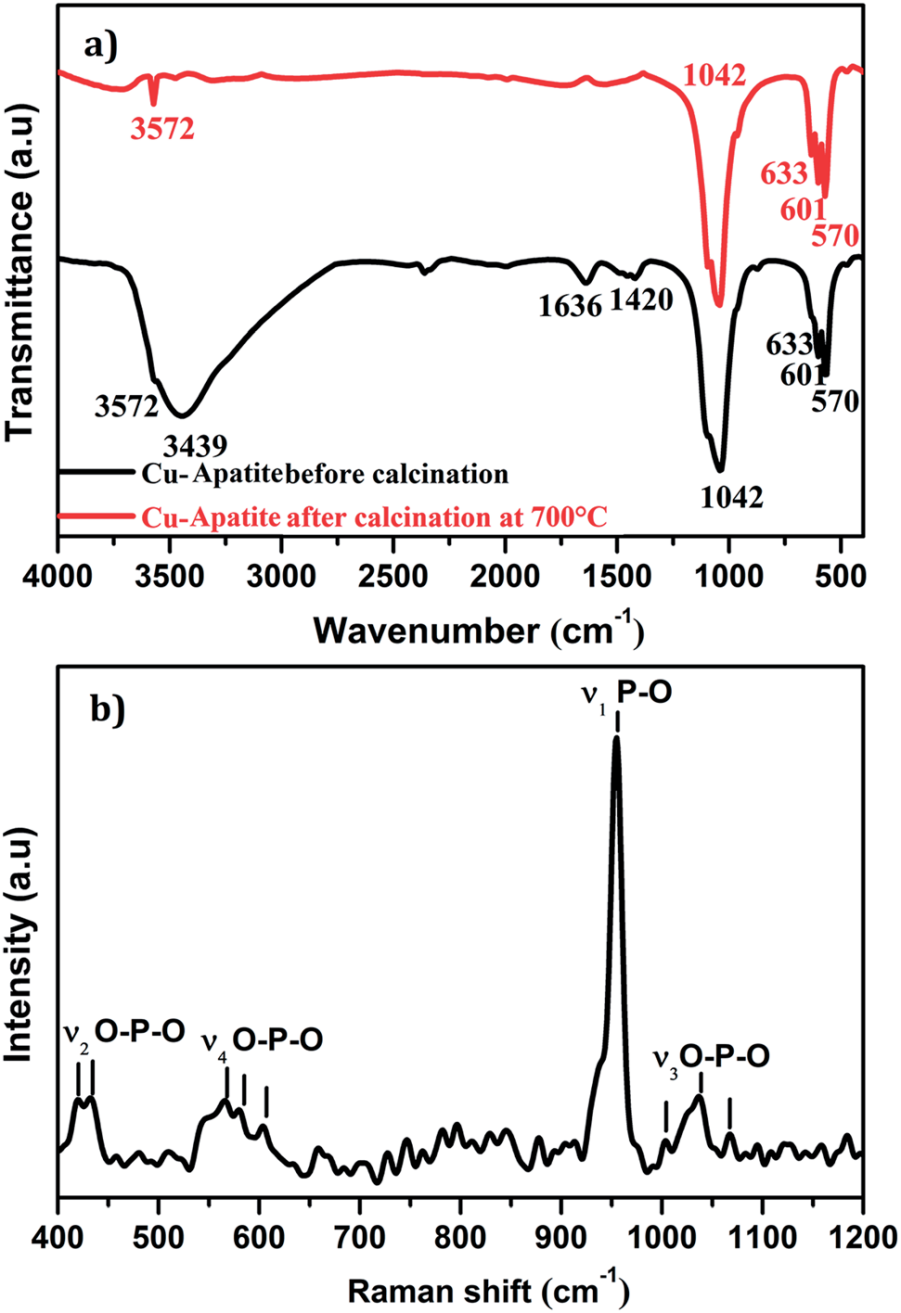
(a) FTIR and (b) Raman shift spectra of Cu-apatite.

Fig. 3a shows FTIR spectra in the range of 400–4000 cm^−1^ of the Cu-apatite before and after heat treatment at 700 °C. As it can be seen, the bands observed at 570 and 601 cm^−1^ are attributed to antisymmetric deformation (*ν*_4_) of PO_4_^3−^ and the intense absorption peak at 1042 cm^−1^ is ascribed to the asymmetric stretching vibration (*ν*_3_) of PO_4_.^3–26^ The peaks located at 3572 and 633 cm^−1^ are attributed to the stretching mode of hydroxyl OH group located in the hydroxyapatite structure. Indeed, the absorption peaks detected at 3439 and 1636 cm^−1^ are corresponding to the bending and the stretching vibrations mode of H_2_O molecules.^27^ Furthermore, the intensity of the peaks located at 1420 cm^−1^ is contributed to the presence of CO_3_^2−^ species in Cu-apatite.^28^ It's worth nothing that all absorption bands corresponding to the vibrations of adsorbed water molecules and carbonate disappeared after the calcination process at 700 °C, which suggests that the organic components have been removed from the Cu-apatite material. Fig. 3b shows the Raman spectrum of the synthesized Cu-apatite. The peaks appearing at ∼1042, 1035 and 1067 cm^−1^ are attributed to the asymmetric stretching mode (*ν*_3_) of the PO_4_ group (P–O bond).^26^ The high intense peak at 954 cm^−1^ corresponds to the symmetric stretching mode (*ν*_1_) of the tetrahedral PO_4_ group (P–O bond).^29^ The modes (*ν*_4_) located at ∼551, 565, 580, and (*ν*_2_) located at ∼418, 437 cm^−1^ can be addressed fundamentally to the bending mode of the PO_4_ group (O–P–O bond).^30^ This results was in agreement with the results obtained from FTIR analysis.

**Fig. 4:**
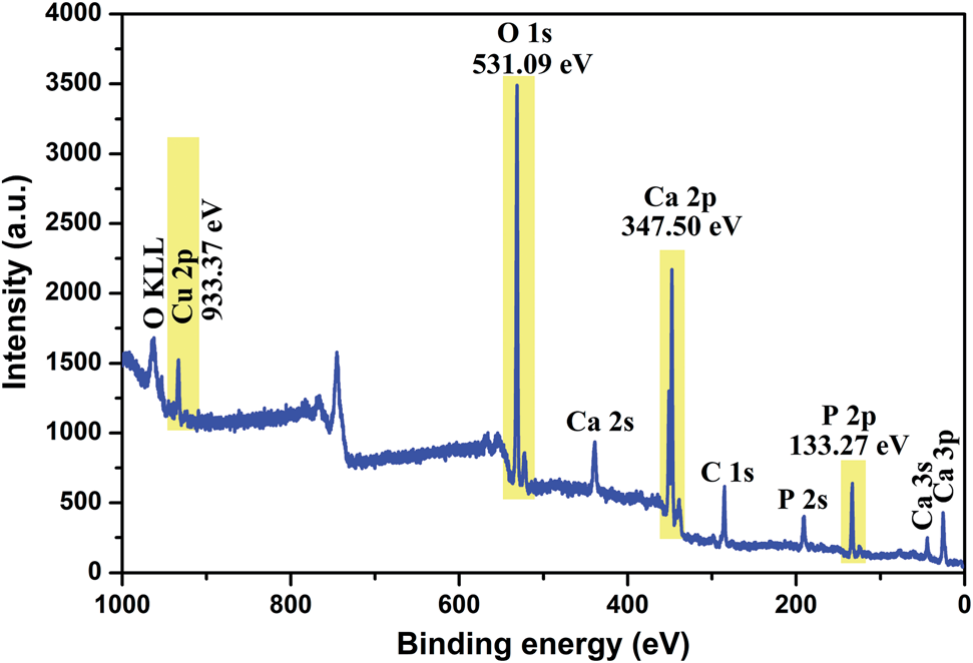
XPS total spectrum of the Cu-apatite catalyst at room temperature.

To investigate the composition and surface chemical states of Cu-apatite, XPS measurement was carried out. Fig. 4 shows the XPS survey spectrum in which it is indicated the presence of principal element namely, Ca 2p, P 2p, O 1s, Cu 2p and C 1s. The presence of a small pic of carbon was due to (CO_3_^2−^) adsorbed from air during synthesis.

**Fig. 5:**
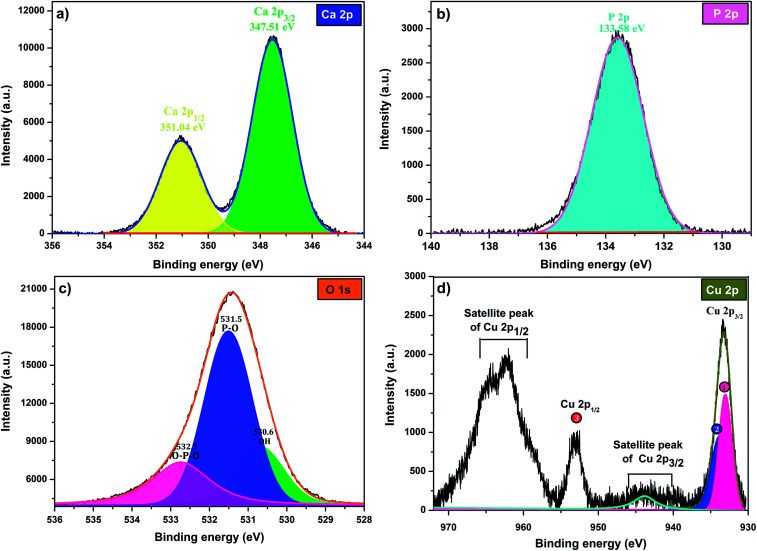
XPS at high-energy resolution on Cu-apatite at room temperature showing: (a) Ca 2p, (b) P 2p, (c) O 1s, and (d) Cu 2p peaks and respective fitting peaks.

The high-resolution XPS scan of Ca 2p as shows (Fig. 5 a) exhibits two peaks Ca 2p_1/2_ at351.4 eV and Ca 2p_3/2_ at 347.51 eV, which are assigned to calcium apatite and Ca–O, respectively.^31^ The binding energy peak at 133.58 eV that fitted with the electron orbit of P 2p_3/2_ 2p is ascribed to the P–O bonds of Hap (Fig. 5b). The XPS spectrum of oxygen O 1 s (Fig. 5c) can be deconvoluted to three characteristic pics at 532.7, 531.5 and 530.6, which could be assigned to O–P–O, P–O and OH, respectively. Indeed, as can be observed in Fig. 5d, the Cu 2p_3/2,_ peak 1 at 933.07 eV is attributed to Cu^2+^/CuO,^32^ while the peak 2 at 933.83 eV is assigned to Cu^+^/Cu_2_O. The formation of Cu^+^ can be explained by the reduction of Cu^2+^ during analysis under X-ray irradiation in ultrahigh vacuum.^33^ The peak Cu 2p_1/2_ at binding energy 953 eV (peak 3) corresponds to Cu^2+^. The peaks positions of the satellites indicate the presence of only Cu^2+^ at the surface of catalyst.

**Fig. 6:**
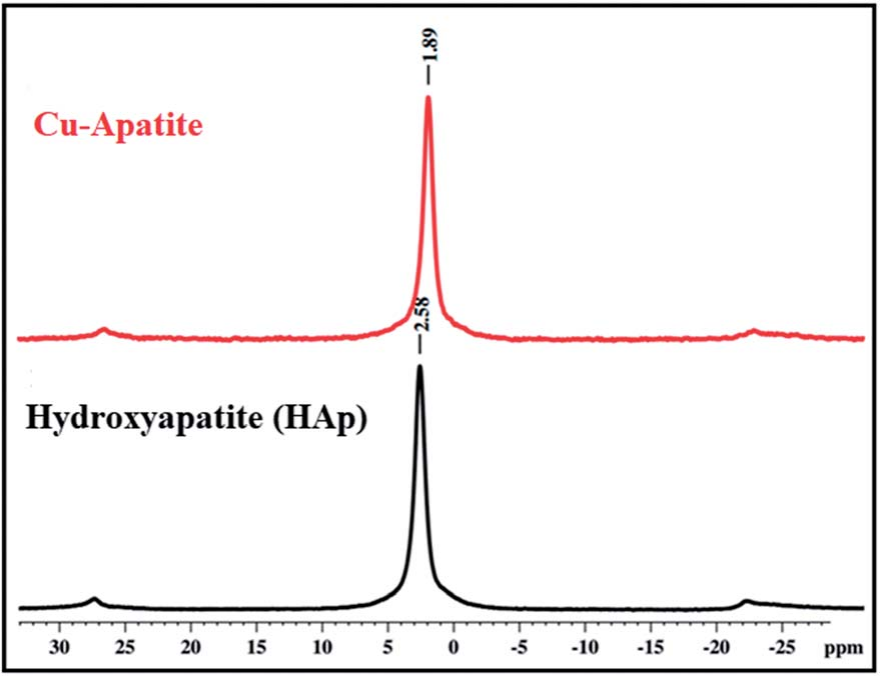
^31^P MAS-NMR spectra of HAp and Cu-apatite.

^31^P solid state NMR was used to investigate the coordination of phosphorus species (PO_4_^3−^) in the prepared hydroxyapatite and Cu-apatite (Fig. 6). The ^31^P MAS NMR spectrum of the hydroxyapatite shows one isotopic signal at 2.58 ppm suggesting the presence of one phosphorus group (PO_4_^3−^) in the prepared HAp.^34^ Moreover, when copper was incorporated to the apatite, the signal of PO_4_^3−^ was shifted from 2.58 to 1.89 ppm.^35^ Indeed, the phosphorus groups cannot be distinguished in the Ca_10_(PO_4_)_6_(OH)_2_ and Ca_19_Cu_2_(PO_4_)_14_ phases by NMR ^31^P, which can be explain by the superposition of the two peaks in the same environment.

**Fig. 7:**
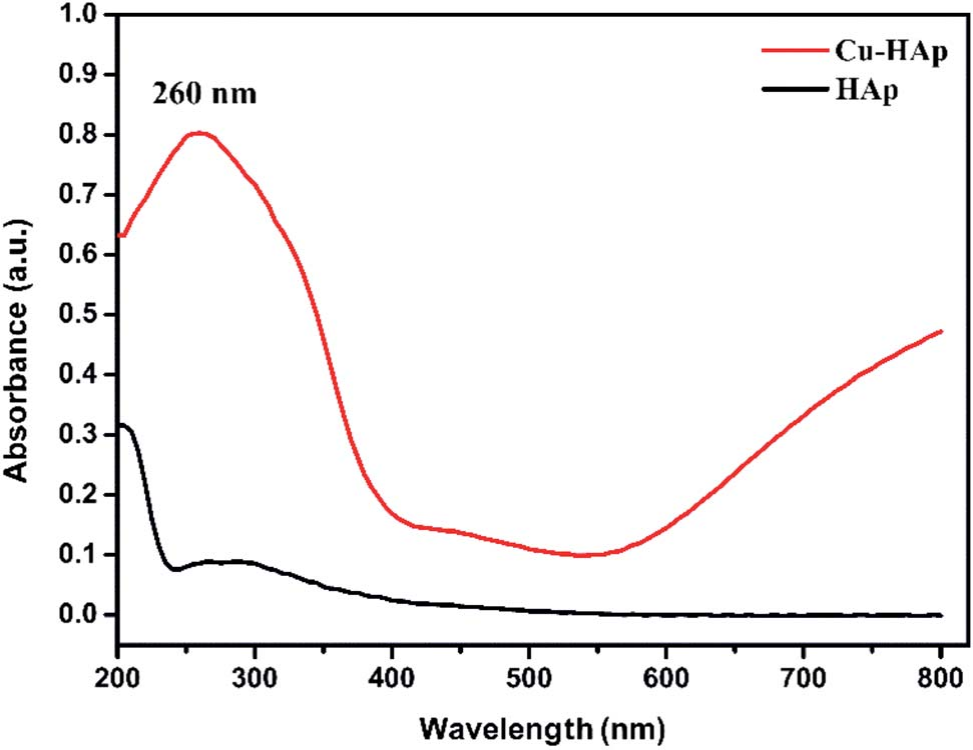
UV-visible profiles of the HAp and Cu-apatite.

The UV-visible diffuse reflectance spectra (DRS) of HAp and Cu-apatite were illustrated in Fig. 7. The pure HAp showed a characteristic peak at 205 nm assigned to O^2−^ → Ca^2+^ charge transfer.^36^ After the incorporation of copper in apatite (Cu-apatite), a new band has appeared at 260 nm attributed to low energy charge transfer O^2−^ → Cu^2+^of copper ions in tetrahedral coordination.^37^ In addition, the increase of intensity of base line in the range of 550–800 identifies the d–d transitions of Cu^2+^ tetrahedral coordination surrounding by oxygen in CuO.

**Fig. 8:**
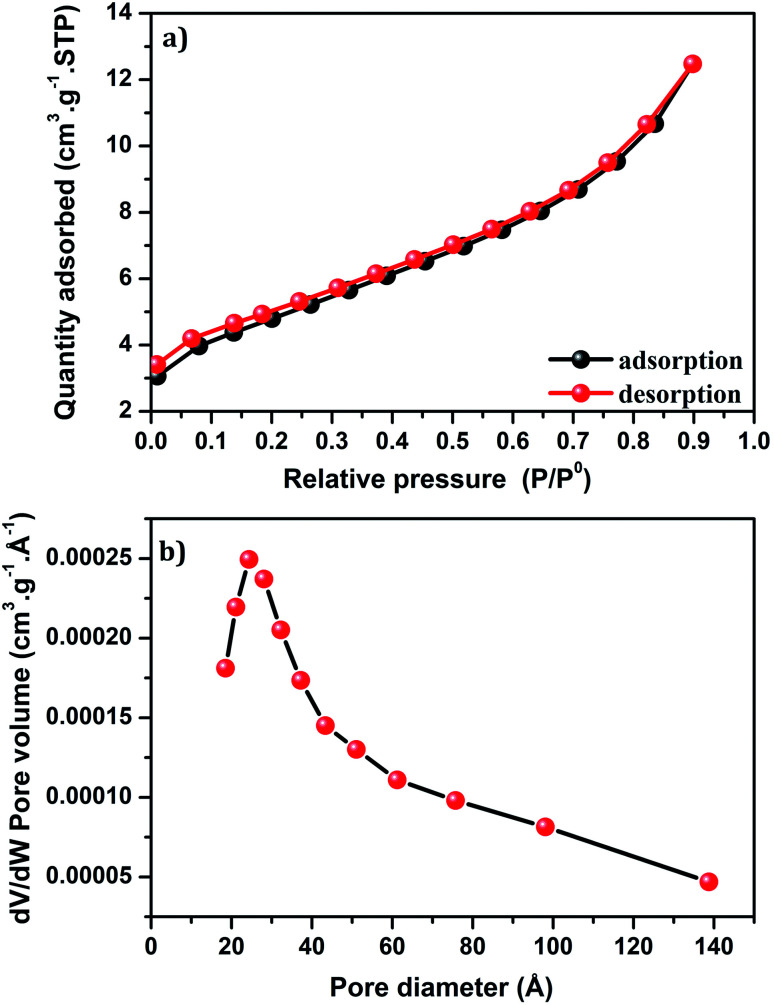
(a) Nitrogen adsorption–desorption isotherm and (b) BJH pore size distribution of Cu-apatite.

Fig. 8a shows the isotherm of Cu-apatite sample. According to the IUPAC classification, the isotherm of Cu-apatite is assigned to type IV with a distinct hysteresis loop of type H3, which is characteristic of mesoporous materials. These results were further confirmed by the pore size distribution curve determined by Barrett–Joyner–Halenda (BJH) method, which showed a centered pore size at 20.43 Å (Fig. 8b). The BET surface area, pore volume and pore diameter of the Cu-apatite material are summarized in Table 1. The specific surface area and the pore volume of the Cu-apatite are found to be 17 m^2^ g^−1^ and 0.00025 cm^3^ g^−1^, respectively (Table 1).

**Table 1:** Textural properties and copper content of Cu-apatite catalyst

Catalyst	Cu^a^ (wt%)	*S*_BET_ b (m^2^ g^−1^)	Pore volume^b^ (cm^3^ g^−1^)	Pore diameter^b^ (Å)
Cu-apatite	1.39	17	0.00025	2.436

a Determined by flame atomic absorption spectroscopy.

b From nitrogen sorption analysis.

**Fig. 9:**
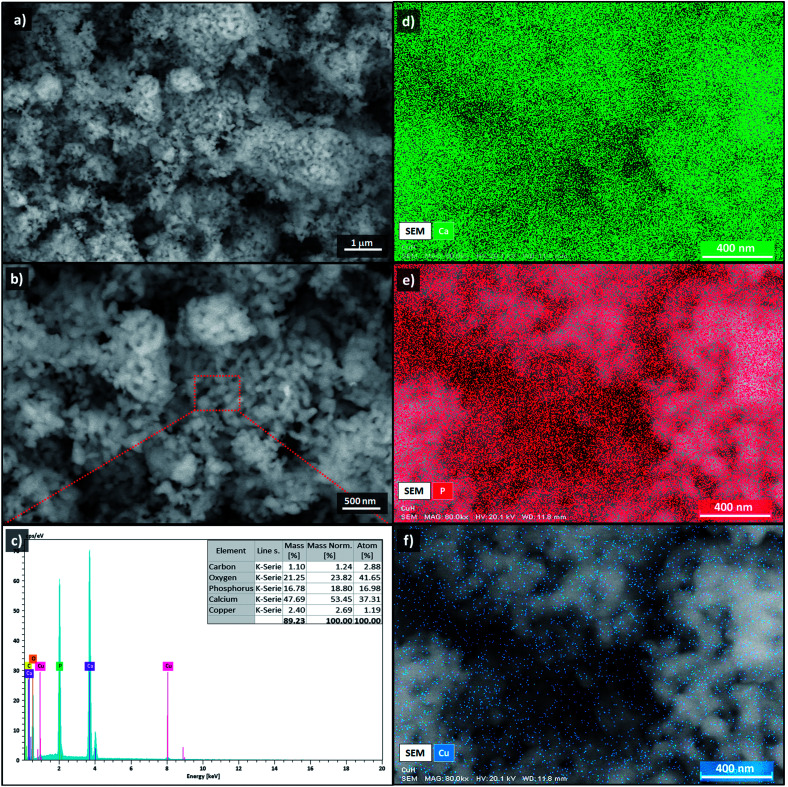
SEM images (a and b), EDS analysis (c) and EDS mapping image (d–f) of Cu-apatite.

The morphology of Cu-apatite was investigated by SEM. As shown in Fig. 9, the Cu-apatite materials had a heterogeneous microstructure that consisted of crystallites with heterogeneous size and forms (Fig. 9a and b). Furthermore, the elemental analysis by energy dispersive spectroscopy (EDS) of Cu-apatite was also performed in different selected zones of the Cu-apatite surface (Fig. 9c). From EDS analysis (Fig. 9d), it was confirmed that Cu-apatite was composed of Ca, Cu, P, O and C. Indeed, the atomic distribution of these elements in the Cu-apatite was Ca = 31.31%, Cu = 1.19%, P = 18.13%, O = 45% and C = 3.89%. The experimental Ca/P molar ratio is equal to 1.73, which is higher than that of theoretical HAp composition (1.67), indicating an even incorporation of Cu in the apatite structure. Moreover, it can be seen, from SEM-EDS maps of Cu-apatite (Fig. 9e–g), that Ca, P and Cu elemental were regularly distributed over the surface of Cu-apatite.

**Fig. 10:**
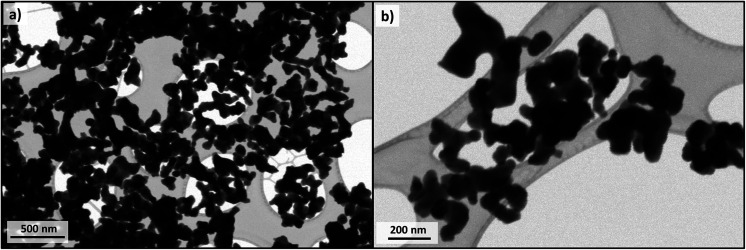
STEM images of Cu-apatite (a and b).

STEM images of Cu-apatite are presented in Fig. 10. According to this figure, the Cu-apatite nanoparticles are well nanostrutured materials with uniform shape and size. We notice also that Cu-apatite dispersion is relatively heterogeneous, since the nanoparticles presented some agglomeration, which can be explained by the Ostwald ripening phenomena.

To evaluate the catalytic performance of Cu-apatite material, the phenol hydroxylation with H_2_O_2_ as oxidant was selected as catalytic reaction mode (Scheme 1). The preliminary experiments were started by screening the activities of hydroxyapatite (HAp) and copper incorporated apatite (Cu-apatite) for catalyst the phenol hydroxylation (Table 2). Firstly, it should be noted that the reaction didn't occur without adding the catalyst (Table 2, entry 1), also the hydroxyapatite was inactive for this reaction (Table 2, entry 2). However, the copper incorporated apatite has high catalytic activity for phenol hydroxylation under the same conditions, indicating that the phenol hydroxylation is very sensitive to the copper species.

**Table 2:** Evaluation and screening of catalysts^a^

Sample	Catalyst	*X*_Ph_ (%)	*Y*_Cat_ (%)	*Y*_HQ_ (%)	*Y*_BQ_ (%)
1	Free catalyst	n.r.	—	—	—
2	HAP	n.r.	—	—	—
3	Cu-apatite	64	68	27	5

a Reaction conditions: phenol (5 mmol); H_2_O_2_ (10 mmol), catalyst (50 mg), water (10 mL), time (2 h) and temperature (60 °C).

**Fig. 11:**
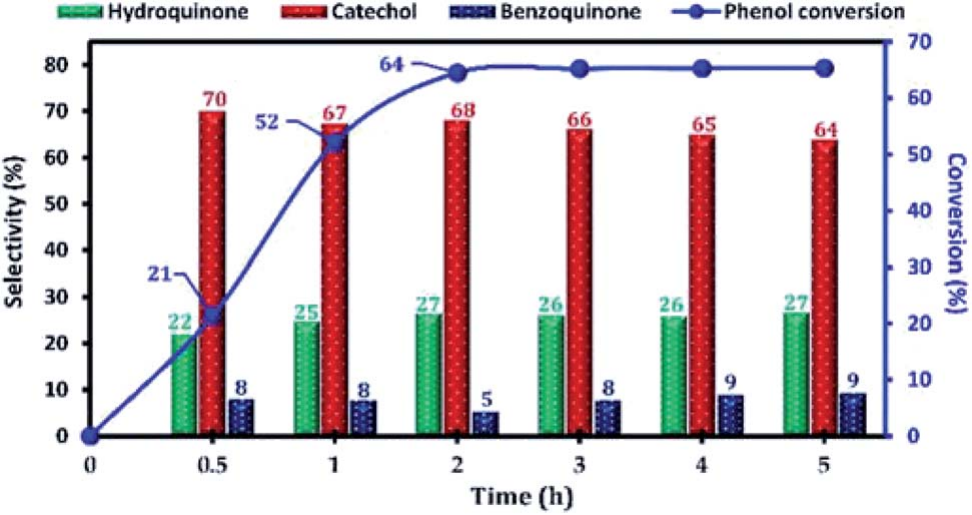
The influence of the reaction time on the phenol hydroxylation over Cu-apatite catalyst. Reaction conditions: phenol (5 mmol), H_2_O_2_ (10 mmol), catalyst (50 mg); water (10 mL), and temperature (60 °C).

The effect of the reaction parameters, namely: reaction time, reaction temperature, H_2_O_2_/phenol molar ratio, type of solvent, catalyst amount, in the phenol hydroxylation reaction was also investigated. Firstly, the influence of the reaction time on the catalytic activity of phenol hydroxylation over Cu-apatite was examined (Fig. 11). From this result, it was found that phenol conversion increases with increasing reaction time. After 2 h, the phenol conversion reached 64% with 95% of dihydroxybenzenes selectivity. However, the increase of time reaction beyond 3 h led to a decrease in the selectivity of hydroquinone, which can be explained by their transformation to the benzoquinone and subsequently to the tar formation.^38^ This indicates that the extension of the reaction time is not desirable for phenol hydroxylation.

**Fig. 12:**
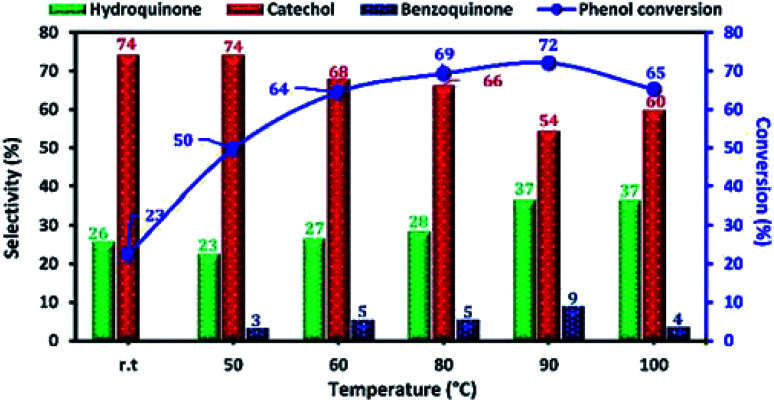
Effect of reaction temperature on phenol hydroxylation over Cu-apatite catalyst. Reaction conditions: phenol (5 mmol), H_2_O_2_ (10 mmol), catalyst (50 mg), water (10 mL) and time (2 h).

The reaction temperature is an important factor affecting the phenol hydroxylation, so we investigated the effect of temperature reaction from 25 to 100 °C. The results of this study are shown in Fig. 12. When the reaction temperature increases from 25 to 90 °C, the phenol conversion increased from 23 to 72%. This result can be explained by the formation of more OH˙ radicals at higher temperatures. However, the presence of a high concentration of free radical led to the oxidation of hydroquinone to benzoquinone. Moreover, when the reaction temperature reached 100 °C (decomposition temperature of H_2_O_2_), the phenol conversion decreased due to the decomposition of H_2_O_2_ to O_2_ and H_2_O.

**Fig. 13:**
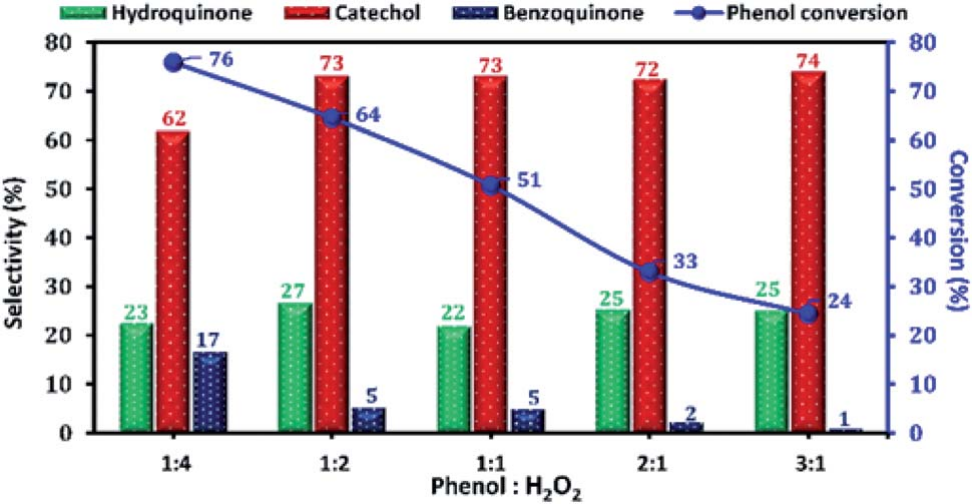
The effect of phenol : H_2_O_2_ molar ratio on phenol hydroxylation over Cu-apatite. Reaction conditions: sample of phenol reacted with H_2_O_2_ in different molar ratios over 50 mg of Cu-apatite in 10 mL of water at 60 °C for 2 h.

The phenol conversion and DHB selectivity over Cu-apatite catalyst was studied by varying the amount of H_2_O_2_ at a fixed amount of phenol. Fig. 13 shows the influence of phenol/H_2_O_2_ molar ratio from 0.25 to 3. From this study, we noticed that the phenol conversion increased from 24 to reach 76% by varying the phenol/H_2_O_2_ molar ratio from 3 : 1 to 1 : 4. This result may be explained by the large generation of OH˙ radicals using an excess amount of H_2_O_2_. However, the excess amount of H_2_O_2_ is undesirable as it decreases the selectivity of DHB. Finally, phenol/H_2_O_2_ molar ratio of 1 : 2 was chosen as the optimum for phenol hydroxylation.

The influence of solvent in the phenol hydroxylation over Cu-apatite catalyst was investigated with various solvent. The results of this study are presented in Table 3. The phenol hydroxylation showed higher conversion and selectivity for DHB when water was used as solvent. However, using methanol, ethanol and acetone as solvent for phenol hydroxylation gives a lower conversion. These results can be explain by the high solubility of phenol and H_2_O_2_ in water and also to the higher stability of free radicals OH˙ in water.^39,40^

**Table 3:** The effect of solvent on the phenol hydroxylation over Cu-apatite catalyst^a^

Solvent	*X*_Ph_ (%)	*Y*_Cat_ (%)	*Y*_HQ_ (%)	*Y*_BQ_ (%)
Water	64	68	27	5
Methanol	5	Trace	Trace	Trace
Ethanol	6	Trace	Trace	Trace
Acetone	3	Trace	Trace	Trace

a Reaction conditions: phenol, 5 mmol; H_2_O_2_, 10 mmol; catalyst (50 mg), solvent (10 mL), time (2 h) and 60 °C.

**Fig. 14:**
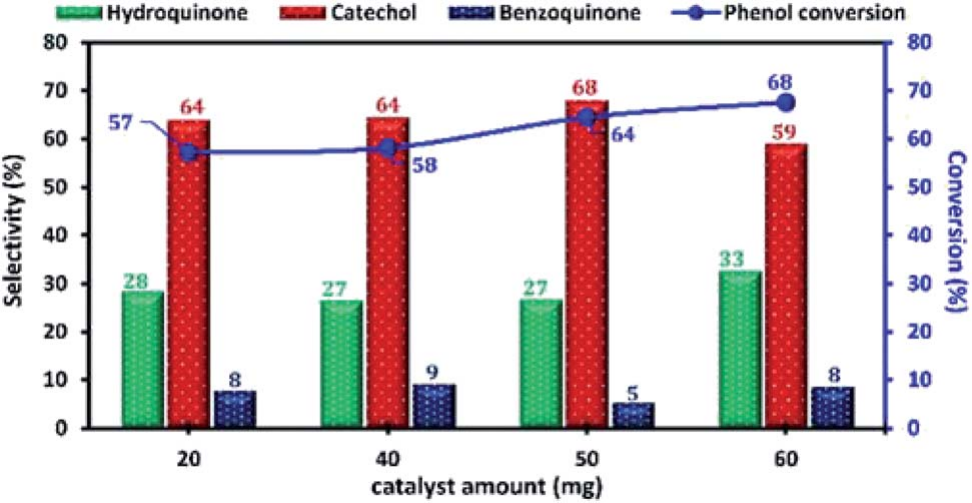
The effect of Cu-apatite catalyst amount on phenol hydroxylation. Reaction conditions: phenol (5 mmol), H_2_O_2_ (10 mmol), water (10 mL), time (2 h), temperature (60 °C).

The influence of the amount of Cu-apatite catalyst ranging from 20 to 60 mg on phenol hydroxylation was also investigated (Fig. 14). As can be seen, the phenol conversion was slightly increasing from 57 to 58% with the increase of the amount of Cu-apatite catalyst from 20 and 40 mg and reached a maximum value when the catalyst amount was increased up to 50 mg. Further increase in Cu-apatite catalyst does not affect significantly the conversion of phenol. Therefore, 50 mg was chosen as the optimal catalyst amount for the phenol hydroxylation.

**Scheme 3:**
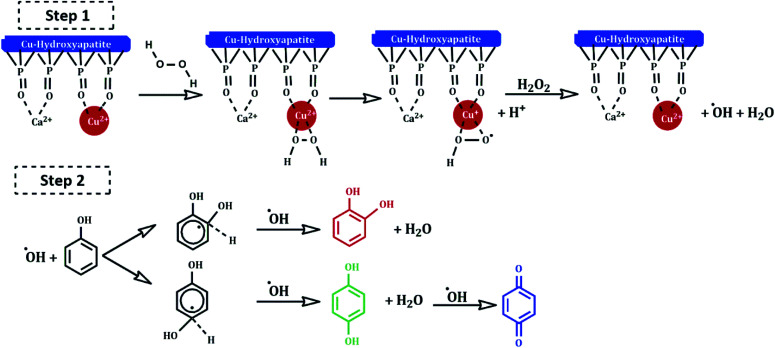
Proposed reaction mechanism of hydroxylation of phenol with H_2_O_2_ catalyzed by Cu-apatite.

Several mechanisms have been proposed in the literature to explain the oxidation of aromatic compounds using transition metals.^41–43^ The plausible reaction mechanism of phenol hydroxylation in the presence of Cu-apatite catalyst is proposed in Scheme 3. Firstly, the catalytic reaction is stated by the reduction of H_2_O_2_ by Cu^2+^-apatite to produce HO_2_˙ and OH˙ radicals *via* a redox mechanism (step 1). Then, the hydroxyl radicals formed oxidize phenol to catechol, hydroquinone and benzoquinone can be obtained by consecutive oxidation of hydroquinone (step 2).

**Fig. 15:**
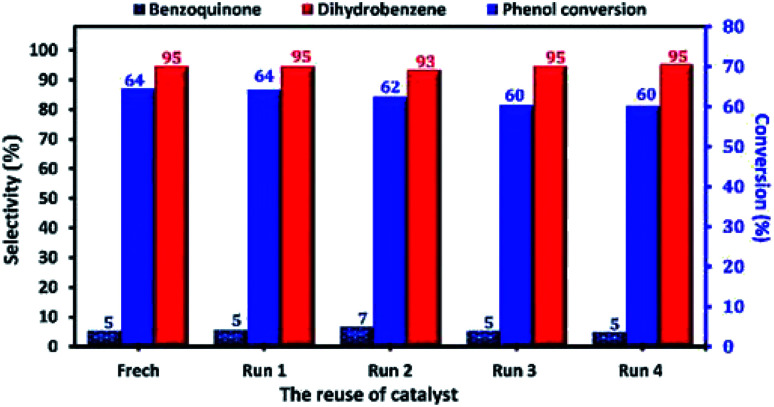
Reuse performance of Cu-apatite catalyst in phenol hydroxylation. Reaction conditions: phenol (5 mmol), H_2_O_2_ (10 mmol), catalyst (50 mg), water (10 mL), time (2 h), temperature (60 °C).

At industrial scale, the reusability of the catalyst is one of the most important parameters of its application as heterogeneous catalyst. Therefore, the reusability and the stability of Cu-apatite in the phenol hydroxylation were investigated (Fig. 15). The regenerated Cu-apatite catalyst was reused for the next run. As shown in Fig. 15, the Cu-apatite demonstrated its ability to be reused for four consecutive cycles with a slight decrease in the activity, which can be due to the gradual poisoning of the surface and pores of Cu-apatite catalyst by tar formation in the phenol hydroxylation.

**Fig. 16:**
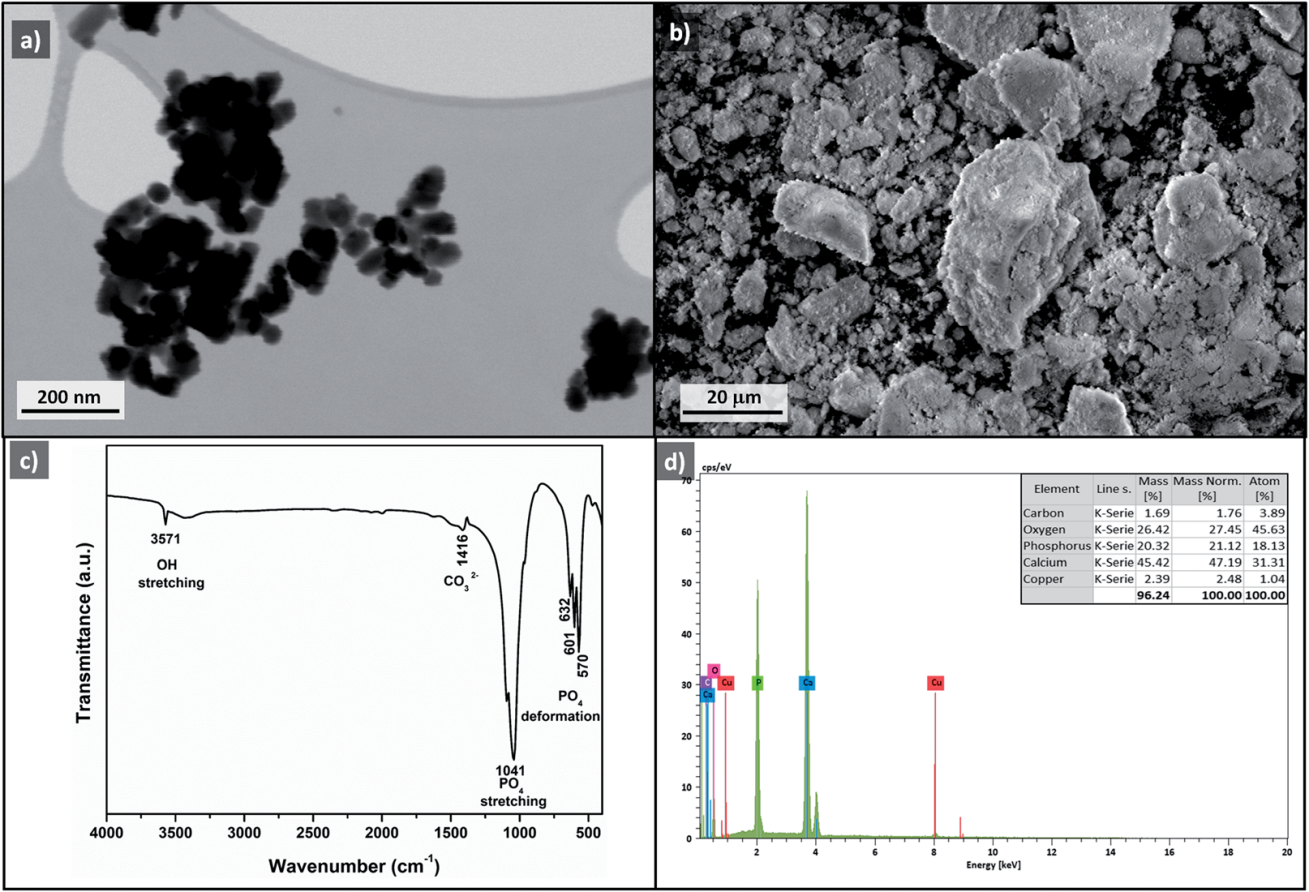
STEM image (a), SEM image (b), FTIR (c) and EDS (d) of Cu-apatite after phenol hydroxylation reaction.

To investigate the heterogeneity of the Cu-apatite catalyst and the copper leaching, the hydroxylation reaction of phenol was maintained for 1 h in the presence of Cu-apatite catalyst. After that, the catalyst was filtered and the filtrate obtained was stirred for additional 1 h at 60 °C, the portion containing the Cu-apatite catalyst showed 52% of phenol conversion, while the no increment of phenol conversion was obtained in the catalyst-free portion, which put in evidence the heterogeneity of the Cu-apatite catalyst. Moreover, copper leaching was studied by atomic absorption spectrometer of the Cu-apatite catalyst after the phenol hydroxylation reaction. The amount of copper incorporated in apatite-based catalyst was found to be 1.24% after one catalytic cycle which confirms no significant copper leaching. The phenol conversion over Cu-apatite was compared with other catalysts reported in the literature as tabulated in Table 4. From this, we can show clearly that our Cu-apatite catalyst exhibited a best catalytic activity of phenol conversion comparing with other catalytic systems. In the other hand, in order to study the stability of Cu-apatite after the phenol hydroxylation reaction, SEM, STEM and FTIR analysis were investigated. The STEM images showed that the size and the form of Cu-apatite nanoparticles remained the same with a heterogeneous dispersion (Fig. 16a). Indeed, the SEM analysis of Cu-apatite after one cycle didn't show any changes in its morphology (Fig. 16b). Also, all absorption bands in the IR spectrum were corresponding to vibrations of original Cu-apatite catalyst (Fig. 16c).

**Table 4:** Comparison of the phenol conversion catalyzed by different catalytic systems

Catalyst	*W*% Fe/Cu	Phenol : H_2_O_2_	Solvent	Temperature (°C)	Time (min)	Phenol conversion (%)	Ref.
Fe/WAC	6.94	1 : 1	Water	40	40	51	44
Cu/MCM-S	1.50	1 : 1	Water	60	360	37	7
Cu-alginate	17.7	1 : 2	Water	70	20	61	45
CuFe_2_O_4_	37.13 (Fe)	1 : 1	Water	55	30	35	46
	21.18 (Cu)	1 : 2			56		
Cu-apatite	1.39	1 : 1	Water	60	120	51	This work
		1 : 2				64	

## 4. Conclusion

Copper incorporated apatite is an active and promising nanocatalyst for high conversion of phenol to high value phenolic compounds. Physicochemical properties of Cu-apatite were evaluated by XRD, FTIR, SEM, STEM, and adsorption–desorption of nitrogen. Moreover, the optimal reaction condition was investigated, and water was proved to be the optimum solvent of the phenol reaction over Cu-apatite. In addition, the Cu-apatite separable catalyst was showed a good catalytic activity with high conversion and selectivity on phenol even after four cycles. The lixiviation study was suggesting negligible copper leaching from Cu-apatite catalyst, which confirms the heterogeneity of our catalyst. Also, the analysis of Cu-apatite recovered catalyst by SEM, STEM and FTIR corroborated the stability of Cu-apatite catalyst.

## Conflicts of interest

The authors declare that there is no conflict of interests regarding the publication of this paper.

## Supplementary Material
